# Baculovirus enhances arginine uptake and induces mitochondrial autophagy to promote viral proliferation

**DOI:** 10.1371/journal.ppat.1013331

**Published:** 2025-07-08

**Authors:** Shigang Fei, Junming Xia, Yigui Huang, Minyang Zhou, Biying Xie, Yibing Kong, Luc Swevers, Jingchen Sun, Min Feng

**Affiliations:** 1 Guangdong Provincial Key Laboratory of Agro-animal Genomics and Molecular Breeding, Guangdong Sericulture Engineering Research Center, College of Animal Science, South China Agricultural University, Guangzhou, China; 2 Hubei Key Laboratory of Edible Wild Plants Conservation and Utilization, Hubei Normal University, Huangshi, China; 3 Insect Molecular Genetics and Biotechnology, Institute of Biosciences & Applications, National Centre for Scientific Research “Demokritos”, Athens, Greece; Institut Pasteur, FRANCE

## Abstract

As obligatory intracellular parasites, viruses must rely on metabolic reprogramming of host cells to meet their replication needs. Baculovirus is an important biopesticide and a vector for the preparation of biological products. In addition, one of its representative species, Bombyx mori nucleopolyhedrovirus (BmNPV-*Baculoviridae*), also causes huge losses to the insect industry. In our previous study, amino acid metabolism has been found to play a crucial role in the BmNPV infection process. However, the mechanisms by which BmNPV reprograms host amino acid metabolism remains unclear. In fact, current insights in the importance of amino acid metabolism are limited to the impact of glutamine on viral infection. Therefore, unraveling the mechanism of amino acid metabolism reprogramming induced by baculovirus would advance this field of research to a great extent. In this study, targeted metabolomics revealed that the preferred amino acids of BmNPV budded virus (BV) include arginine, lysine, proline, isoleucine, histidine and others. In addition, most of the viral amino acids were found to be increased in the hemolymph of BmNPV infected silkworms at the later stage of infection, especially arginine, valine, phenylalanine and others. Furthermore, the importance of arginine for BmNPV proliferation was validated. Next, we confirmed that the expression of the arginine transporter Slc7a6 was strongly induced by BmNPV infection and that Slc7a6 could promote arginine uptake to support BmNPV proliferation in host cells. Moreover, using Slc7a6 knockout cells which eliminate extracellular arginine uptake, we confirmed that BmNPV could induce mitochondrial autophagy, thereby supplementing intracellular arginine and providing necessary amino acids for BmNPV proliferation. Overall, these findings support a model in which baculovirus (BmNPV) enhances the uptake of exogenous amino acids by inducing the expression of amino acid transporters and activating autophagy of organelles to maintain intracellular amino acid levels, thereby facilitating virus proliferation.

## Author summary

Baculovirus is an important biopesticide and a vector for the preparation of biological products. In addition, one of its representative species, Bombyx mori nucleopolyhedrovirus, also causes huge losses to the insect industry. However, the mechanisms by which BmNPV reprograms host amino acid metabolism remains unclear. Therefore, unraveling the mechanism of amino acid metabolism reprogramming induced by baculovirus would advance this field of research to a great extent. In this study, our findings revealed that the predominant amino acids of BmNPV budded virus include arginine, lysine and proline. In addition, most of the viral key amino acids were found to be increased in the hemolymph of BmNPV infected silkworms at the later stage of infection, especially arginine. Furthermore, the importance of arginine for BmNPV proliferation was validated. Next, we confirmed the molecular mechanism by which BmNPV supplements intracellular arginine levels to maintain virus replication through the induction of arginine transporter Slc7a6 expression and mitochondrial autophagy. Overall, our data support a model whereby baculoviruses establish an “exogenous uptake-endogenous supply” paradigm by synergistically enhancing external nutrient acquisition and activating autophagy-mediated amino acid replenishment. This dual mechanism maintains intracellular amino acid homeostasis to facilitate baculovirus proliferation. Moreover, it reveals the viral strategy of hijacking host amino acid transport and the cellular autophagy system for efficient amino acid exploitation, offering a new perspective on host-virus interactions.

## Introduction

As obligate intracellular parasites, viruses lack an independent metabolic system and therefore rely entirely on host cell resources to obtain the chemical building blocks required for their proliferation and replication [1–3]. Upon infecting host cells, viruses employ various mechanisms to coerce, hijack, and exploit host metabolic pathways to supply essential materials and energy for progeny virus production [3,4]. During this process, several host metabolic pathways, including amino acid, nucleotide, lipid, and energy metabolism, undergo significant alterations [3]. For instance, Newcastle disease virus modulates host nucleotide and glutamine metabolism to facilitate its own replication [5,6], while African swine fever virus enhances its replication efficiency by regulating host energy and amino acid metabolism [7].

Amino acids are crucial intracellular metabolites that play a role in the synthesis of bioactive molecules and in critical processes such as signal transduction and metabolic regulation [8–10]. Consequently, maintaining amino acid homeostasis is essential for cell survival. The regulation of intracellular amino acid levels in host cells involves amino acid transport mediated by amino acid transporters (AAT), intracellular biosynthesis, recycling of damaged organelles, and protein degradation [11,12]. Viruses typically exploit the host’s amino acid resources to facilitate their own production, thereby impacting host amino acid metabolism. However, whether viruses exhibit specific preferences for certain amino acids, and which mechanisms are used to reprogram host cell metabolism to regulate amino acid availability, remain subjects for further investigation.

Bombyx mori nucleopolyhedrovirus (BmNPV) is an enveloped, double-stranded circular DNA virus belonging to the *Baculoviridae* family [13]. Infection with BmNPV in silkworms leads to symptoms such as hyperactivity, swelling and tissue liquefaction leading to the formation of milky hemolymph, ultimately resulting in host death, and therefore posing a significant threat to the sericulture industry [14–16]. The genome of BmNPV is approximately 130 kb in size and contains around 136 predicted open reading frames [17,18]. Upon infecting the host, BmNPV requires substantial consumption of host metabolites, such as nucleotides and amino acids, to support its replication. Furthermore, studies have shown that amino acid metabolism-related pathways are significantly enriched following BmNPV infection [19–21]. Therefore, amino acid metabolism might play a crucial role during BmNPV infection. However, questions such as how host cells meet the amino acid demands for viral protein synthesis and which mechanisms are employed to regulate host amino acid metabolism remain to be further explored.

In this study, targeted metabolomics were employed to analyze the amino acid composition and content of BmNPV virions and the reprogramming effects of BmNPV infection on host amino acid metabolism. The results indicated that arginine was a preferential amino acid to promote the replication of BmNPV. Furthermore, our data revealed that BmNPV effectively maintains intracellular arginine levels through a dual mechanism, which included upregulating the expression of the arginine transporter *Slc7a6* and inducing host autophagy.

## Results

### Amino acid profiling of the BmNPV budded virus (BV)

**Fig 1:**
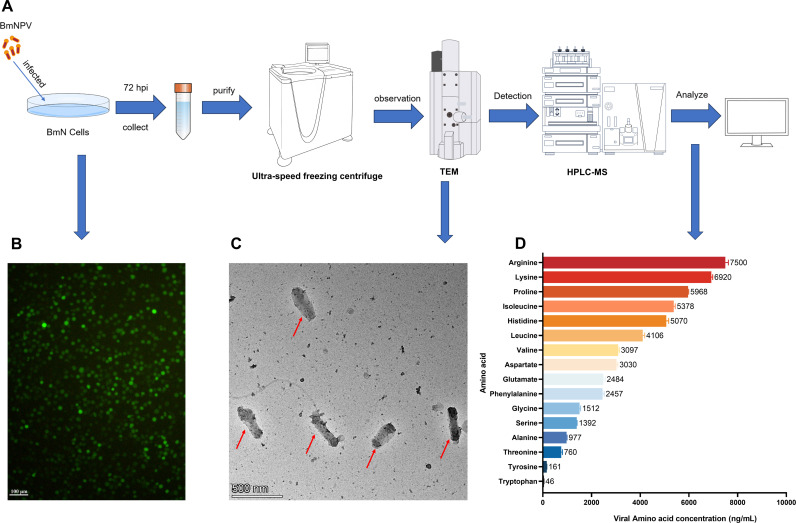
Amino acid composition content of BmNPV. (A) Flow chart of BmNPV purification and amino acid detection. (B) Green fluorescence was observed 72 h after BmN cells were infected with BmNPV-eGFP. Scale bar: 100 μm. (C) Examination of purified BmNPV using transmission electron microscopy (TEM). Red arrows: Purified virus particles (BV) (D) Amino acid composition content of BmNPV.

To understand the amino acid composition of BmNPV, we purified and assayed BmNPV-eGFP virions according to the flowchart in Fig 1A. When BmN cells were infected with BmNPV-eGFP, intense green fluorescence was observed after 72 h (Fig 1B), indicating successful viral propagation and protein expression. The culture medium of infected BmN cells was used for BmNPV purification by ultracentrifugation using a discontinuous sucrose gradient. It was observed that the BmNPV virus particles in the samples were intact and that the background was relatively free of impurities (Fig 1C). The size of the purified BmNPV virions was consistent with the literature (typically 40 nm–60 nm in diameter and 240 nm–390 nm in length [22] (Fig 1C). Targeted amino acid metabolism analysis of BmNPV-eGFP virions showed that the 10 most abundant amino acids in BmNPV included arginine, lysine, proline, isoleucine, histidine, leucine, valine, aspartic acid, glutamic acid, and phenylalanine which were all essential amino acids for silkworms (Fig 1D). Most notably, arginine is the most abundant amino acid in BmNPV BV virus particles (Fig 1D).

### BmNPV reprograms amino acid metabolism in the silkworm and arginine promotes BmNPV proliferation

**Fig 2:**
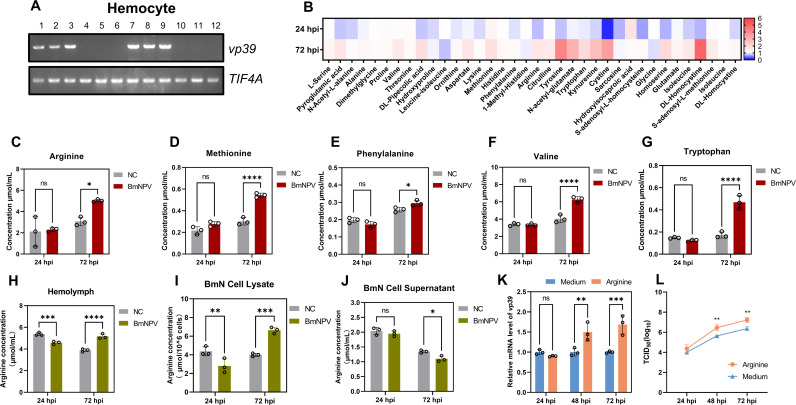
BmNPV reprograms amino acid metabolism in the silkworm and arginine promotes BmNPV replication in BmN cells. (A) Hemocytes were collected at 24 h and 72 h after BmNPV injection into the silkworm and the viral gene *vp39* was detected using RT-PCR using *TIF4A* as a reference gene. 1-3: Hemocytes infected with BmNPV for 24 h; 4-6: Hemocytes uninfected with BmNPV for 24 h; 7-9: Hemocytes infected with BmNPV for 72 h; 10-12: Hemocytes uninfected with BmNPV for 72 h. (B) Targeted amino acid metabolomics was used to analyze changes in amino acid abundance in the hemolymph of the silkworm at 24 h and 72 h after BmNPV infection. (C-G) Changes of arginine (C), methionine (D), phenylalanine (E), valine (F), and tryptophan (G) in the hemolymph of BmNPV-infected silkworms at 24 and 72 h. (H) Changes of arginine content in the hemolymph of BmNPV-infected silkworms, at 24 and 72 hpi. (I, J) The concentration of arginine in BmN cells (I) and BmN cell supernatants (J) was measured at 24 h and 72 h after BmNPV infection of BmN cells. (K, L) After pretreatment of BmN cells with 3 mM arginine for 12 h and incubation of cells with 1 MOI of BmNPV, cell and supernatant samples were collected at 24, 48, and 72 hpi. The mRNA level of the viral gene *vp39* was detected using qPCR (K) and the viral TCID_50_ was determined (L). Each bar represents the mean ± SD. *P < 0.05, **P < 0.01, ***P < 0.001, ****P < 0.0001. ns, not significant. NC, negative control.

To understand the dynamic changes of amino acid abundance in the hemolymph of BmNPV-infected silkworms, BmNPV-eGFP was injected into silkworm larvae and hemolymph samples were collected at 24 and 72 hpi. Subsequently, the expression level of viral capsid gene *vp39* in the hemocyte samples was detected by RT-PCR successfully (Fig 2A). Heatmap visualization from targeted amino acid metabolomics was used to analyze amino acid changes in hemolymph samples, which showed that most of them exhibited a decreasing trend at 24 hpi and an increasing trend at 72 hpi compared to the control (Fig 2B).

The levels of essential and non-essential amino acids during BmNPV infection were analyzed by targeted metabolomics. The levels of arginine (Fig 2C), methionine (Fig 2D), phenylalanine (Fig 2E), valine (Fig 2F), and tryptophan (Fig 2G) increased significantly in the hemolymph at 72 hpi after BmNPV infection. However, methionine was not detected in the amino acid targeted metabolome analysis of BV amino acid content (Fig 1D). In addition, the contents of most of the nonessential amino acids such as alanine, tyrosine, glycine, serine and 3-phospho-serine also increased significantly at 72 hpi, with sarcosine, N-acetyl-L-alanine and hydroxyisocaproic acid as exceptions (S1 Fig). These results suggested that BmNPV infection could induce reprogramming of host amino acid metabolism to facilitate its own proliferation.

Arginine had the highest concentration in BmNPV virions (Fig 1D), and there was a significant change in the arginine content in the hemolymph after viral infection (Fig 2C). Therefore, arginine was selected for further analysis of its regulation during infection. The detection of arginine in the hemolymph of BmNPV-infected and uninfected silkworms by biochemical assay showed that the arginine content in the hemolymph of BmNPV-infected silkworms was significantly reduced at 24 hpi, but significantly increased at 72 hpi (Fig 2H). This result was consistent with the results of amino acid targeted metabolomics analysis in the hemolymph (Fig 2C).

Similarly, in BmNPV-infected BmN cells, the arginine content decreased significantly at 24 hpi and increased significantly at 72 hpi (Fig 2I). By contrast, the arginine content in the cellular medium was significantly decreased at 72 hpi (Fig 2J). Next, to explore the effect of arginine on BmNPV replication, arginine was added to the culture medium and the cells and supernatants were collected at 24, 48 and 72 h, respectively, to detect the replication of BmNPV. The results showed that the addition of arginine significantly increased the mRNA level of *vp39* (Fig 2K) as well as the viral titer of BmNPV, as measured by the TCID_50_ assay (Fig 2L). The same experiment also confirmed that another constituent amino acid (top 12) of BV, serine (Fig 1D), could promote BV proliferation in BmN cells at 48 and 72 hpi (S2A and S2B Fig). These results suggested that the constituent amino acids of virions could indeed promote the proliferation of the virus.

### Arginine transporter Slc7a6 increases intracellular arginine intake and promotes BmNPV proliferation

**Fig 3:**
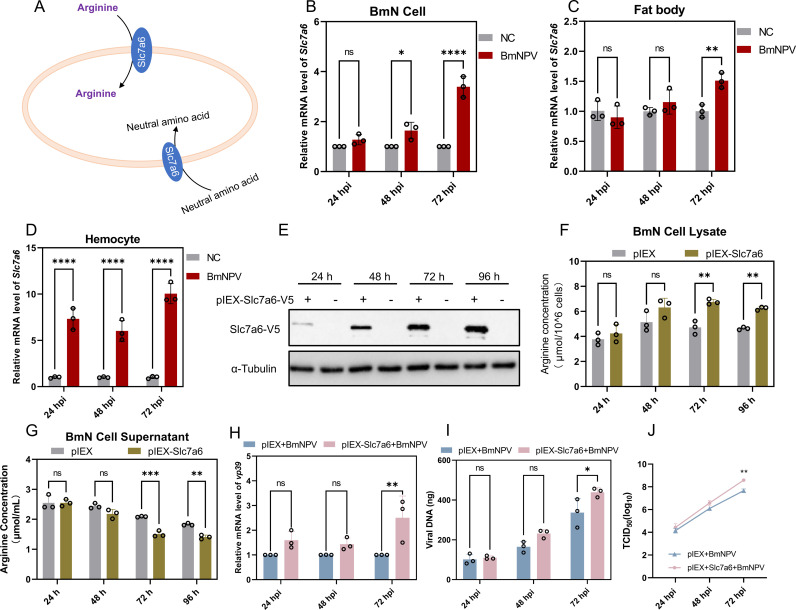
BmNPV infection induces significant up-regulation of Slc7a6 to promote self-replication. (A) Schematic diagram of amino acid transport by Slc7a6. Slc7a6 participates in the transport of cationic and large neutral amino acids. (B-D) BmN cells (B), fat body tissue (C), and hemocytes (D) were collected at 24, 48, and 72 h after infection with BmNPV, and the expression levels of the *Slc7a6* gene were detected by qPCR. (E) BmN cells were seeded on a 24-well plate, and when the cell density reached 70-80%, 500 ng of pIEX-Slc7a6-V5 was transfected into BmN cells, and the same concentration of pIEX served as a control. Cell samples were collected at 24, 48, 72 and 96 h after transfection. The expression level of Slc7a6 was detected by Western blot using V5 antibody. (F, G) After pIEX-Slc7a6-V5 was transfected into BmN cells, cells (F) and supernatants (G) were collected at 24, 48, 72, and 96 h and assayed for the concentration of arginine. (H**,** I) BmN cells were transfected with pIEX-Slc7a6-V5 for 24 h. After 24 h, BmN cells were incubated with 1 MOI of virus for 1 h and then washed and cultured with fresh Grace’s medium, and cell samples were collected at 24, 48, and 72 hpi. The mRNA level of the viral gene *vp39* (H) and the viral DNA load (I) were detected respectively. (J) Detection of viral titers using the TCID_50_ assay. Each bar represents the mean ± SD. *P < 0.05, **P < 0.01, ***P < 0.001, ****P < 0.0001. ns, not significant.

As an essential amino acid for silkworms, arginine cannot be synthesized at sufficient quantities and therefore needs to be assimilated from the food. We therefore analyzed the amino acid transporters of *Bombyx mori* using the previously determined hemocyte transcriptome [19] and found that the amino acid transporter gene *Slc7a6* was significantly up-regulated in BmNPV-infected hemocytes. Slc7a6 is capable to function as a transporter of arginine (Fig 3A) [23]. To further explore the response of *Slc7a6* to virus infection, the expression level of *Slc7a6* was determined in BmNPV-infected BmN cells, which showed a significant up-regulation at 48 and 72 hpi (Fig 3B). In fat body, the expression of *Slc7a6* was significantly induced by BmNPV infection at 72 hpi (Fig 3C). In addition, we found that *Slc7a6* expression was significantly up-regulated in BmNPV-infected hemocytes at 24, 48 and 72 hpi (Fig 3D). Obviously, the response of the *Slc7a6* to viral infection implied its potential role in the process of BmNPV replication through arginine transport (Fig 3A-D). Therefore, the effect of Slc7a6 on arginine content inside and outside the cell, and BmNPV replication was further verified after overexpression of Slc7a6 in BmN cells.

Western blot analysis showed that Slc7a6-V5 was successfully expressed in BmN cells (Fig 3E), which was accompanied by a significant increase in the intracellular arginine content at 72 and 96 h post-transfection (Fig 3F). Conversely, the arginine content in the cellular medium was significantly reduced at 72 and 96 h post-transfection (Fig 3G). When BmN cells were infected with BmNPV at 24 h after transfection of *Slc7a6*, it was observed that the mRNA expression level of the viral gene *vp39* (Fig 3H), the viral DNA load (Fig 3I), and the titer of the virus (Fig 3J) were both significantly up-regulated at 72 hpi. These results suggested that overexpression of Slc7a6 could enhance the cellular uptake of arginine to benefit BmNPV proliferation.

### Knocking down of *Slc7a6* reduces intracellular arginine content and inhibits BmNPV replication

**Fig 4:**
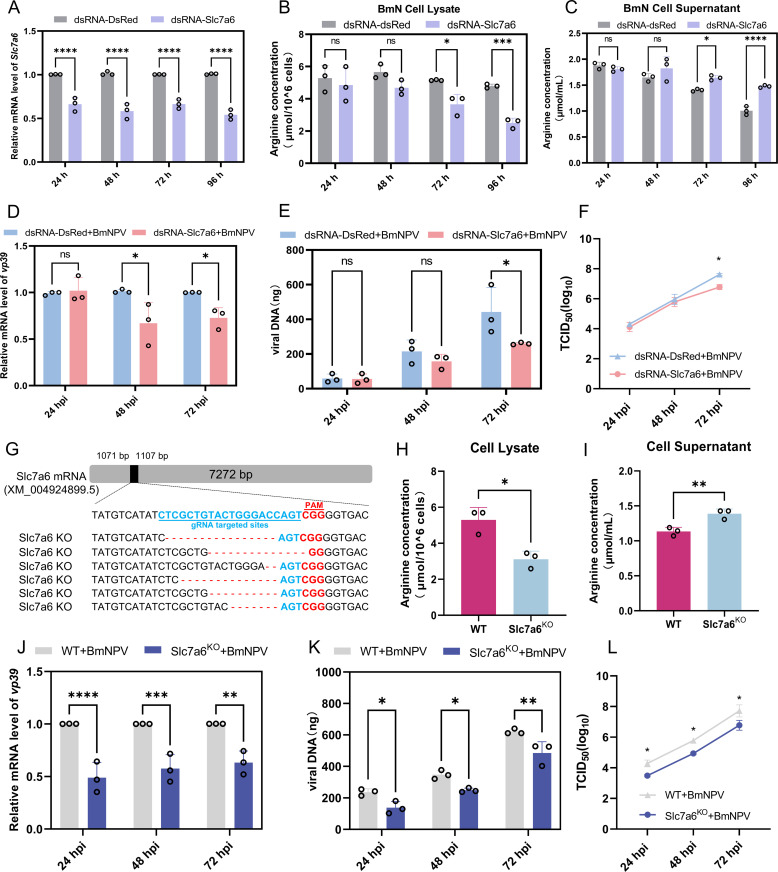
Knockdown of Slc7a6 reduces intracellular arginine content and inhibits BmNPV replication. (A) BmN cells were seeded on a 24-well culture plate, 5 μg of dsRNA-Slc7a6 was transfected into BmN cells, and cell samples were collected at 24, 48, 72, and 96 h to detect the knockdown efficiency of Slc7a6. (B, C) After transfection of BmN cells with the same concentration of dsRNA-Slc7a6, cell (B) and supernatant (C) fractions were harvested at 24, 48, 72, and 96 h post-transfection, respectively, to assay arginine concentration. (D, E) At 24 h after BmN cells were transfected with dsRNA-Slc7a6, BmN cells were incubated with 1 MOI of BmNPV for 1 h. Subsequently, the cells were washed with PBS and incubated with fresh Grace’s medium. At 24, 48 and 72 hpi, cell samples were collected and the mRNA level of viral gene *vp39* (D) and the viral DNA load (E) were detected by qPCR. (F) Supernatants were collected 24, 48 and 72 h after BmNPV infection of cells and viral titers were assayed using the TCID_50_ assay. (G) Sequence analysis of the targeted sequence in WT and Slc7a6^KO^ BmN cell lines. (H, I) Of the Slc7a6^KO^ and WT BmN celllines, cells (H) and the corresponding supernatants (I) were collected to assay the arginine content. (J, K) Slc7a6^KO^ cells were inoculated in 24-well cell culture plates and subsequently infected with 1 MOI of BmNPV virus. At 24, 48 and 72 hpi, cell samples were collected and tested for mRNA levels of the viral gene *vp39* (J) and the viral DNA load (K) using qPCR. (L) Detection of supernatant titers 24, 48 and 72 h after BmNPV infection of Slc7a6^KO^ cells using the TCID_50_ assay. Each bar represents the mean ± SD. *P < 0.05, **P < 0.01, ***P < 0.001, ****P < 0.0001. ns, not significant.

After transfection of dsRNA-Slc7a6, BmN cell samples showed significant suppression of *Slc7a6* mRNA compared with the control group (Fig 4A), together with a significant drop in intracellular arginine content and a significant increase in extracellular arginine in the cellular medium at 72 and 96 h after transfection of dsRNA-Slc7a6 (Fig 4B and 4C). Moreover, knockdown of *Slc7a6* in BmN cells and subsequent infection with BmNPV revealed a significant decrease in virus *vp39* expression (48 and 72 hpi), viral DNA load (72 hpi), and viral titer (72 hpi) in the later stages of infection (Fig 4D, 4E and 4F).

To further validate the effect of *Slc7a6* on arginine content as well as BmNPV proliferation, Slc7a6^KO^ BmN cells were generated by CRISPR/Cas9 technology (Figs 4G, S3A and S3B). After transfection of pSL1180-Cas9-U6-Slc7a6, surviving BmN cells were selected by Zeocin and further analyzed for expression levels of *Slc7a6* (S3A and S3B Fig). Sequence analysis showed that the ORF of *Slc7a6* was successfully deleted by 2–16 nucleotides (Fig 4G), presumably corresponding to different cellular subpopulations. Additionally, the cell viability showed no difference from that of normal cells (S3C Fig).

When analyzed for intracellular arginine content, its levels were found to be significantly reduced in the Slc7a6^KO^ cell line compared with the control group (Fig 4H), while the arginine content in the cellular medium of the Slc7a6^KO^ group was significantly up-regulated (Fig 4I). Obviously, the absence of Slc7a6 significantly affected the cellular uptake of arginine. As observed for silencing of Slc7a6 with dsRNA, deficiency in intracellular arginine inhibited BmNPV infection in Slc7a6^KO^ cells, illustrated by quantification of *vp39* mRNA, viral DNA load assessment, and viral titer measurements (Fig 4J, 4K and 4L). The difference is that in Slc7a6^KO^ cells, each detection time point of 24, 48 and 72 hpi showed significant inhibition of virus proliferation, rather than 48 or 72 hpi in RNAi knockdown cells (Fig 4D,4E,4F,4J, 4K and 4L). These results suggest that inhibition of Slc7a6 expression attenuated cellular uptake of arginine and inhibited BmNPV proliferation.

### Autophagy rescues intracellular amino acid content and promotes BmNPV proliferation in Slc7a6^KO^ cells

**Fig 5:**
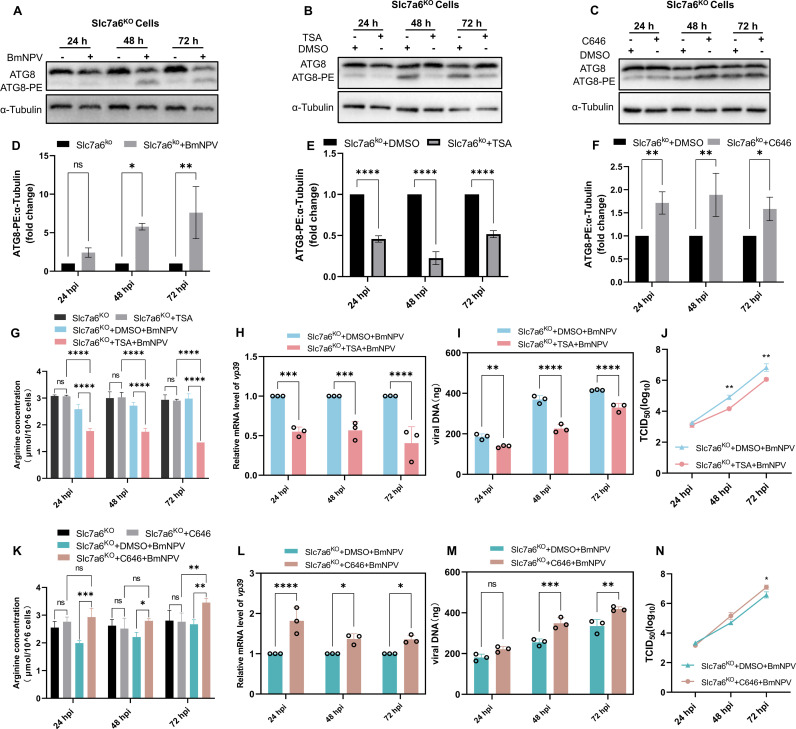
Effect of autophagy on amino acid content and BmNPV replication in Slc7a6^KO^ cells. (A) Western blotting was used to detect ATG8-PE in Slc7a6^KO^ cells after BmNPV infection. (B, C) After pretreatment of Slc7a6^KO^ cells with 4 nM TSA (B) or 100 nM C646 (C) for 12 h, the cells were incubated with 1 MOI of BmNPV, followed by incubation of the cells with fresh medium containing 4 nM TSA (B) or 100 nM C646 (C). Cells were collected at 24, 48 and 72 hpi, and Western blotting was used to detect ATG8-PE in Slc7a6^KO^ cells. (D) Quantification of ATG8-PE in Panel A. (E) Quantification of ATG8–PE in Panel B. (F) Quantification of ATG8-PE in Panel C. (G, K) Uninfected and BmNPV-infected Slc7a6^KO^ BmN cells were treated with 4 nM TSA (G) or 100 nM C646 (K). Cell samples collected at 24, 48 and 72 hpi were assayed for the concentration of arginine. (H**,** I) After pretreating Slc7a6^KO^ cells with 4 nM TSA for 12 h (DMSO was used as a control), the cells were incubated with 1 MOI of BmNPV, followed by incubation with fresh medium containing 4 nM TSA. Cells were collected at 24, 48 and 72 hpi to detect changes in mRNA expression levels of the viral gene *vp39* (H) and the viral DNA load (I). (J) Detection of viral titer by TCID_50_. (L, M) Slc7a6^KO^ BmN cells were pretreated with 100 nM C646 for 12 h (DMSO was used as a control). At the end of pretreatment, cells were infected with 1 MOI of BmNPV virus and continued in fresh medium containing 100 nM C646. At 24, 48 and 72 hpi, cell samples were collected and examined for changes in mRNA expression levels of the viral gene *vp39* (L) and the viral DNA load (M). (N) Detection of viral titer by TCID_50_. The Western blotting results are representative of one of three independently performed. Each bar represents the mean ± SD. *P < 0.05, **P < 0.01, ***P < 0.001, ****P < 0.0001. ns, not significant.

To investigate the role of autophagy in amino acid metabolism during BmNPV infection, Slc7a6^KO^ cells were used to minimize the influence of exogenous arginine uptake. We first confirmed that BmNPV infection could also induce autophagy in Slc7a6^KO^ cells (Fig 5A and 5D). That is, 48 and 72 h after BmNPV infection, the conversion of ATG8 to ATG8-PE was enhanced in Slc7a6^KO^ cells (Fig 5A and 5D). Next, the autophagy inhibitor TSA and autophagy inducer C646 were used to treat the Slc7a6^KO^ cells. Using toxicity assays, the optimal concentrations of TSA (S4A Fig) and the C646 (S4B Fig) were determined as 4 nM and 100 nM, respectively. Subsequently, BmNPV-infected Slc7a6^KO^ cells were treated with TSA or C646, and the occurrence of autophagy in the cells was examined. The results showed that TSA was able to inhibit BmNPV-induced conversion of ATG8 to ATG8-PE in Slc7a6^KO^ cells (Fig 5B and 5E), indicating that TSA suppressed BmNPV - induced autophagy in Slc7a6^KO^ cells. Conversely, the use of C646 enhanced autophagy (Fig 5C and 5F). Next, the concentration of arginine in the cells was assayed following treatment with TSA (Fig 5G) or C646 (Fig 5K). Treatment of uninfected Slc7a6^KO^ cells with TSA or C646 had no effect on intracellular arginine content compared to untreated controls (Fig 5G and 5K). On the other hand, after 24 h of infection with BmNPV in Slc7a6^KO^ cells, the intracellular arginine was significantly decreased (Figs 5G, 5K and S3D), similar to wild-type BmN cells (Fig 2I). After infection with Slc7a6^KO^ cells for 48 and 72 h, the levels of arginine in the Slc7a6^KO^ cells were replenished, especially at 72 hpi, which was basically the same as the control group (Figs 5G, 5K and S3D). However, unlike the significant upregulation of intracellular arginine in wild-type BmN cells at 72 hpi (Fig 2I), there was no upregulation of intracellular arginine in Slc7a6^KO^ cells after 72 h of BmNPV infection, which may be due to the inability of Slc7a6^KO^ cells to take up arginine from the culture medium (Figs 5G,5K and S3D). These results indicated that during infection of Slc7a6^KO^ with BmNPV at 48 and 72 hpi, certain mechanisms within the cells restored amino acid balance, which is likely due to virus induced autophagy. Treatment with autophagy inducers and inhibitors confirmed this assumption. After treating BmNPV - infected cells with the autophagy inhibitor TSA, the intracellular arginine content decreased significantly (Fig 5G). Conversely, treating Slc7a6^KO^ cells with the autophagy inducer C646 led to a significant increase in intracellular arginine levels at 24–72 h post - BmNPV infection, compared to untreated controls (Fig 5K). Moreover, the autophagy inhibitor TSA was found to inhibit BmNPV proliferation in Slc7a6^KO^  cells (Fig 5H, 5I and 5J) while autophagy inducer C646 could promote of BmNPV proliferation (Fig 5L, 5M and 5N). These results suggested that BmNPV infection induced cells to initiate autophagy, which in turn replenishes the intracellular arginine required to maintain their proliferation.

### Effect of BmNPV-induced mitochondrial autophagy on intracellular arginine levels

**Fig 6:**
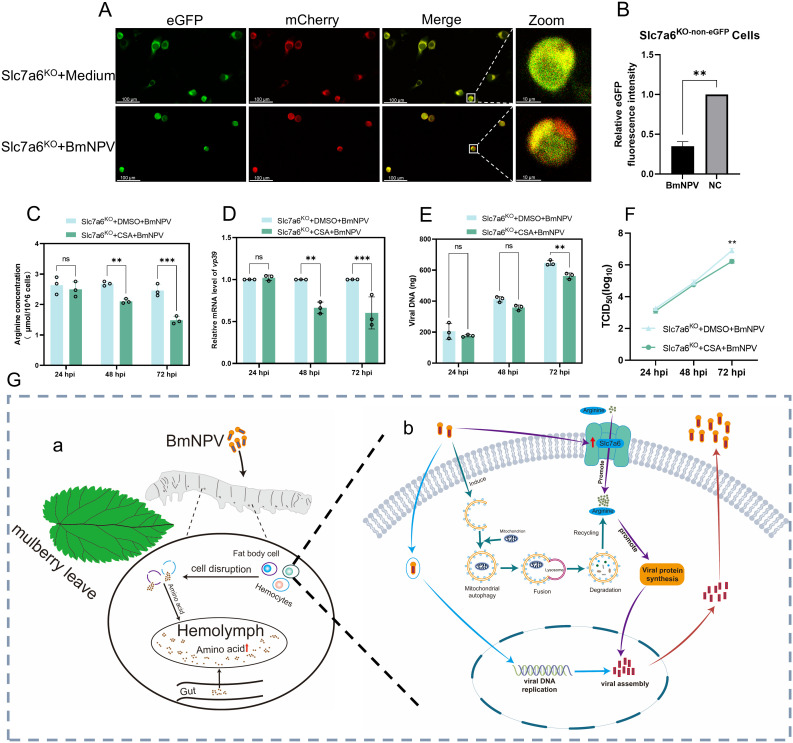
Supplementation of intracellular arginine by mitochondrial autophagy benefits BmNPV replication. (A) The Mito-QC reporting system detects mitochondrial autophagy in BmNPV-infected Slc7a6^KO-non-eGFP^ cells. Scale bar: 100 μm and 10 μm. (B) Calculation of green fluorescence intensity of mitochondria in Slc7a6^KO-non-eGFP^ cells using FiJi software. (C) Slc7a6^KO^ cells were pretreated with 30 nM of CSA for 12 h (DMSO was used as a control) followed by infection with 1 MOI of BmNPV. Cell samples were collected at 24, 48, and 72 hpi to assay for arginine content. (D, E) Slc7a6^KO^ cells were pretreated with CSA for 12 h and Slc7a6^KO^ cells followed by infection with 1 MOI of BmNPV. Cell samples were collected at 24, 48 and 72 hpi to detect the mRNA level of the viral gene *vp39* (D) and the viral DNA load (E), respectively. (F) The supernatant of CSA-treated BmNPV-infected cells at 24, 48, and 72 h was collected for viral titer detemination using the TCID_50_ method. Each bar represents the mean ± SD. *P < 0.05, **P < 0.01, ***P < 0.001. ns, not significant. (G) Overview of the mechanisms by which BmNPV maintains intracellular arginine levels to benefit self-replication. Panel a display the processes in silkworm larvae: both hemocyte and fat body cell disruption because of BmNPV infection and nutrient transport from the gut epithelium after feeding contribute to the increase of amino acid levels in the hemolymph. Panel b shows the regulation of the levels of the amino acids in individual cells: induction of mitochondrial autophagy and transmembrane transport of by Slc7a6 result in an increase of cytosolic arginine levels.

Our previous study found that mitochondrial morphology was altered and mitochondrial function was impaired after BmNPV infection of BmN cells [24]. Furthermore, we used the Mito-QC system to detect whether BmNPV infection leads to mitochondrial autophagy. The results showed that the mitochondrial green fluorescent signal was significantly reduced in BmNPV-infected wild-type BmN cells compared with normal BmN cells (S5A and S5B Fig), indicative of quenching of green fluorescence that occurs during delivery to lysosomes by mitophagy [25]. Furthermore, the Mito-QC system confirmed that BmNPV infection of Slc7a6^KO-non-eGFP^ cells also induces mitophagy (Fig 6A and 6B).

To investigate whether mitochondrial autophagy affects intracellular arginine content, we used the mitochondrial autophagy inhibitor, Cyclosporine A (CSA) [26]. At the optimal CSA concentration (30 nM; S4C Fig), the content of arginine was significantly reduced in the Slc7a6^KO^ cells at 48 and 72 hpi (Fig 6C). More importantly, after treating  Slc7a6^KO^ cells with mitochondrial autophagy inhibitor CSA, the proliferation of BmNPV showed a significant decrease in the middle and late stages of infection at the level of *vp39* expression (Fig 6D), the viral DNA load (Fig 6E), and the viral titer (Fig 6F).

These results suggested that BmNPV induces cellular mitochondrial autophagy to replenish intracellular arginine, thereby favoring its replication.

## Discussion

Amino acids, as essential cellular biomolecules, are crucial for maintaining metabolic balance. Studies have shown that viral infections can significantly impact the amino acid metabolism of host cells. For example, Kaposi’s sarcoma-associated herpesvirus infection induces host cells to increase the uptake and breakdown of glutamine [27]. Similarly, cells infected with Epstein-Barr virus also show enhanced glutamine uptake [28]. In this study, we analyzed the changes in amino acids in silkworm hemolymph following infection by BmNPV using targeted metabolomics. The results showed that in the early stage of infection (24 hpi), amino acid levels in the hemolymph generally decreased, possibly reflecting the consumption of host cell amino acids during viral replication. However, as the infection progressed to later stages (Fig 2), amino acid levels in the hemolymph began to recover. A possible explanation could be the increased uptake from the external environment by silkworm feeding, triggered by the need to maintain normal metabolism. During virus infection, this need becomes exacerbated since high amounts of amino acids are consumed for the production of virions.

For cells, during the early stages of BmNPV infection, the virus consumes large amounts of amino acids from the host cell, leading to a decrease in intracellular amino acid levels. By 72 hpi, the peak period of viral replication, BmNPV may reprogram the host’s amino acid metabolism pathways to replenish intracellular amino acid content, thereby restoring or even increasing amino acid levels within the cells.

For individual silkworms, we speculate that the massive proliferation of the virus in the later stages of BmNPV infection can lead to excessive cell rupture and consequent release of amino acids into the hemolymph, thereby further increasing the amino acid content in the hemolymph. This was also reflected in our previous studies of BmNPV-infected hemocytes and fat body, where we found a decrease in the number of hemocytes and fat body cells 72 h after infection with BmNPV [29,30]. During the infection period, the silkworm keeps on feeding, resulting in nutrient uptake from the gut and transport of amino acids in the hemolymph, where it can be distributed to internal tissues [31]. The combination of these two processes (cell lysis and uptake of nutrients) may be responsible for the increase in amino acid content in the hemolymph at the late stage of BmNPV infection (Fig 6G Panel a). In the present study, expanding on the characteristics of the amino acid composition of the baculovirus virions and the changes in hemolymph amino acids after infection, the mechanism of host cell amino acid reprogramming at the level of the individual cell was also revealed and included (1) upregulating the expression of amino acid transporters to facilitate extracellular uptake of amino acids, and (2) inducing (mitochondrial) autophagy to supplement intracellular amino acid consumption (Fig 6G Panel b).

However, apart from glutamine, the roles of the other amino acids in supporting viral replication have not been thoroughly investigated. Glutamine, as the most abundant amino acid in mammals, not only provides a carbon source for host cells but also serves as a substrate for synthesizing other amino acids [32]. Interestingly, metabolic studies of BmNPV infection have found that glutamine metabolism appears to be minimally affected by BmNPV infection [19,20]. In our study, no glutamine was detected in silkworm hemolymph (Fig 2), nor was glutamine detected in the amino acid composition analysis of BmNPV virions (Fig 1).

In silkworms, 13 essential and 5 non-essential amino acids were determined [33,34]. This indicates that most amino acids required by silkworms cannot be synthesized internally and must be obtained from the food. Notably, in the amino acid composition of BmNPV virions, the top ten amino acids are all essential for silkworms, with arginine being the most abundant (Fig 1). Additionally, at 72 h post-BmNPV infection, the level of arginine in the hemolymph significantly increased (Fig 2), and therefore represented an extracellular source to support for BmNPV replication. Because of its abundance in virions and its significant increase in the hemolymph following infection, arginine was considered as a representative amino acid to explore how BmNPV regulates host cell metabolism to meet its amino acid requirements.

Regrettably, the unavailability of amino acid-deficient medium poses a significant drawback. Such a medium would have been highly advantageous for investigating amino acid metabolism in BmN cells. In subsequent experiments, we added arginine (3 mM) to Grace’s medium (i.e., doubling the dose since the concentration of arginine in Grace’s medium is 3 mM; Thermo Fisher, technical resources) and found that arginine supplementation promoted BmNPV replication (Fig 2K and 2L). Furthermore, the trend of arginine levels in BmN cells after BmNPV infection was consistent with the changes observed in hemolymph (Fig 2H,2I, and 2J). The decline of arginine in the supernatant of infected BmN cells at 72 hpi could be explained by its consumption by the cells, in contrast to the hemolymph, where amino acids are expected to be continuously replenished by the digestion of the food. As arginine is an essential amino acid for silkworms 33, 34], amino acid transporters play a key role in this process. Additionally, in the transcriptome analysis of BmNPV-infected silkworm hemolymph, the amino acid transporter gene *Slc7a6* was found to be significantly upregulated [19]. Further experimental results demonstrated that BmNPV induced upregulation of *Slc7a6* in hemocytes, fat body and BmN cells (Fig 3B,3C, and 3D). This suggests that BmNPV may utilize Slc7a6 to maintain intracellular amino acid levels, and we further confirmed this result (Figs 3F-J and 4).

In mammals, Slc7a6 functions as the light subunit in the heterodimeric transport system y^+^L, that transports cationic amino acids (which includes arginine but also lysine) and large neutral amino acids, and for which Slc7a7 can be used as an alternative [35,36]. In addition, other members of the solute carrier superfamily can also transport cationic acids (e.g., Slc7a1, Slc7a2 and Slc7a3) [37]. It is noted that Slc7a6 is especially highly expressed in macrophages [35] for which the hemocytes are the functionally equivalent cells in insects [38, 39]. Thus, in particular cell types, Slc7a6 may be a limiting factor for transmembrane transport of arginine. Interestingly, expression of Slc7a6 is associated with the development of hepatocellular carcinoma [40,41], presumably by providing sufficient amounts of amino acids necessary for cell growth, which was also proposed for other Slc members [36]. Correspondingly, the expression level of Slc7a6 was significantly upregulated in liver tumor cells, which enhances arginine uptake [42]. Thus, a limiting role for Slc7a6 in the regulation of growth or proliferation is not unprecedented and could be extended to virus replication and virion production in the silkworm/BmNPV infection model. In *Drosophila*, Slc genes also have been implicated in the regulation of growth [43]. Mammalian *Slc7a6* together with *Slc7a7* are considered orthologous to *genderblind* which controls synapse strength and sexual courtship in flies [44].

Regarding the mechanism by which BmNPV infection could induce the expression of Slc7a6, little information is available. Because of the challenge of baculovirus infection, it can be assumed that the integrated stress response is activated, as has been documented for mammalian viruses [45,46]. A key regulator in the stress response is Activating Transcription Factor 4 (ATF4) that induces a wide range of genes, including those related to amino acid metabolism [47]. Induction of ATF4 has been detected during infections of several mammalian viruses such as cytomegalovirus, herpes virus, hepatitis B virus and hepatitis C virus [48–51]. Of note, ATF4 has been shown to regulate the expression of the cationic amino acid transporter Slc7a5 as well as other amino acid transporters [52]. It can therefore be assumed that BmNPV manipulates the stress response for the provision of amino acids during the infection process, but this will require further investigation. Research on the regulation of the cellular stress response during baculovirus infection has mainly focused on the prevention of apoptosis and the role of heat shock proteins [53,54] while limitations in amino acid availability have not received much attention.

The intracellular source of amino acids relies not only on external uptake but also on the recycling pathways through degradation of cellular components [55,56]. Autophagy is an evolutionarily conserved process, from unicellular eukaryotes to primates, that maintains cellular homeostasis [57,58]. During autophagy, damaged organelles and macromolecules are degraded, generating a new supply of amino acids, nucleotides, and other nutrients, which are subsequently re-utilized by the cell [59]. Our previous research showed that BmNPV infection induces autophagy in BmN cells, which supports viral replication [24]. Therefore, we investigated whether the virus increases intracellular amino acid levels via autophagy. The results showed that BmNPV was able to utilize autophagy to increase intracellular arginine levels and promote viral replication (Fig 5).

In general, viral infections usually result in damage to host organelles, which in turn triggers organelle-specific autophagy. For example, Newcastle disease virus induces cellular autophagy in specific organelles such as mitochondria and endoplasmic reticulum to promote replication [60–62]. Mitochondrial autophagy likewise has an active role in hepatitis C virus infection [63]. However, reports on viruses inducing the onset of organelle autophagy and thus replenishing intracellular nutrients such as amino acids are relatively rare. In our study, we found that mitochondrial autophagy played an important role in maintaining amino acid homeostasis in BmN cells during BmNPV infection (Fig 6A-6F). Whether BmNPV infection triggers the occurrence of autophagy in other organelles has not been reported, and will be a focus for future research.

In summary, our findings indicate that BmNPV infection reprograms amino acid metabolism in cells. Our data, for the first time, reveal that BmNPV sustains intracellular arginine supply to support viral replication through the “exogenous uptake-endogenous supply” model: by upregulating the expression of the amino acid transporter *Slc7a6* and by inducing autophagy (Fig 6G Panel b). These discoveries do not only deepen our understanding of the amino acid requirements during BmNPV replication but also uncover the viral strategy of integrating host transmembrane transport and cellular autophagy for efficient amino acid exploitation, offering a new perspective on host-virus metabolic interactions.

## Materials and methods

### Cell line, silkworm strain, expression plasmid and recombinant BmNPV

The silkworm BmN cell line was cultured in Grace’s medium, supplemented with 10% Fetal Bovine Serum (FBS, Gibco, USA), at 28°C in a humidified incubator. Silkworm larvae (*Bombyx mori*, Dazao P50 strain) were reared under conditions of 28°C and 70–80% relative humidity. The over-expression plasmid pIEX-his was obtained from Wuhan Miaoling Biotech Co., Ltd. The recombinant BmNPV-eGFP virus was stored in the Key Laboratory of Agricultural Genomics and Molecular Breeding in Guangdong Province.

### Targeted metabolomics: sample preparation and detection

Silkworms were injected with 10 μL of BmNPV-eGFP (TCID_50_ = 10^5.8^/0.1 mL) at day 1 of the 5th instar, while the control group was injected with an equivalent volume of sterile water. At 24 and 72 hours post-infection (hpi), hemolymph samples from both the treatment and control groups were collected and processed following the method described by Feng *et al*. (2021a), with each sample comprising hemolymph from six silkworms and each group having three replicates [19]. Subsequently, the supernatant samples (after removal of hemocytes) were rapidly frozen in liquid nitrogen and stored at -80°C. After the successful infection of the samples with BmNPV was confirmed by RT-PCR, targeted metabolomics analysis was subsequently performed on the hemocytes. Specifically, 100 μL of hemolymph was aliquoted into 2 mL centrifuge tubes, followed by the addition of 800 μL of 80% methanol containing an internal standard at a concentration of 100 ng/mL. The mixture was vortexed for 1 min, homogenized for 5 min, and sonicated at 4°C for 30 min. Thereafter, the samples were incubated at 60°C for 10 min and centrifuged at 12,000 rpm for 10 min. The supernatant obtained post-centrifugation was used for subsequent instrumental analysis. Targeted metabolomics sequencing analysis was conducted by Gene Denovo Biotechnology Co. (Guangzhou, China).

### Ultra-high performance liquid chromatography mass spectrometry (UPLC-MS)

UPLC-MS analysis was conducted using a Waters UPLC BEH Amide chromatographic column (1.7 µm, 2.1 mm × 100 mm) interfaced with a mass spectrometry system. The mobile phase was composed of water (solvent A) and acetonitrile (solvent B). The elution gradient was as follows: from 0 to 1 minute, 85% B; from 1 to 9 min, a linear gradient from 85% to 55% B; from 9 to 10 min, isocratic at 55% B; from 10 to 11 min, a linear gradient from 55% to 85% B; and from 11 to 12 min, isocratic at 85% B. The column temperature was maintained at 40°C, and the sample injection volume was set to 6 μL.

For the mass spectrometry conditions, electrospray ionization (ESI) was employed. The curtain gas flow rate was set at 35 arbitrary units (arb), the collision gas flow rate at 7 arb, the ion spray voltage at 4500 V, and the ion source temperature at 450°C. The flow rates for both ion source gas 1 and ion source gas 2 were maintained at 55 arb. Then, the amino acids in the samples were identified according to the chromatographic and mass spectrometric conditions of each amino acid standard (Shanghai yuanye Bio-Technology, China), and the content of the amino acids was calculated.

### Determination of the amino acid content in BmNPV

BmN cells were evenly seeded onto 10 cm^2^ cell culture dishes and infected with BmNPV-eGFP at a multiplicity of infection (MOI) of 1. At 72 hpi, after successful observation of cell fluorescence, 300 mL of cell supernatant was collected for virus purification. The purification of BmNPV was achieved through discontinuous sucrose gradient ultracentrifugation at 45,000 g for 6 hours at 4°C. The purified BmNPV samples were examined by transmission electron microscopy to confirm their quality before proceeding to amino acid composition analysis. Briefly, the samples were ground into a powder under liquid nitrogen and then lyophilized. To the lyophilized samples, 1.5 mL of 6 M HCl was added, and the samples were hydrolyzed at 110°C for 24 h. After cooling to room temperature, the pH was adjusted to neutrality with a sodium hydroxide solution, and the volume was made up to 1 mL. The resulting solution was filtered through a 0.22 μm aqueous filter membrane and stored at -20°C until analysis on the instrument.

The analysis was conducted using a high-performance liquid chromatography (HPLC) system (Agilent 1260) interfaced with a triple quadrupole mass spectrometer (Agilent 6420A). The mobile phase consisted of water adjusted to pH 3 (solvent A) and 90% acetonitrile (solvent B). The chromatographic separation was achieved with the following gradient elution program: 95% B from 0 to 0.01 min; a linear gradient from 95% to 80% B from 0.01 to 4 min; isocratic at 80% B from 4 to 6 min; a linear gradient from 80% to 55% B from 6 to 12 min; a linear gradient from 55% to 95% B from 12 to 12.1 min; and isocratic at 95% B from 12.1 to 17 min. The sample injection volume was 1 μL, and the detection wavelength was set at 254 nm. For the mass spectrometric analysis, electrospray ionization (ESI) in positive ion mode with multiple reaction monitoring (MRM) was employed. The source parameters included a gas temperature of 350°C, a gas flow rate of 10 L/min, a nebulizer pressure of 45 pounds per square inch (psi) and capillary voltages of 4000 V for positive ions and 3500 V for negative ions. Then, the amino acids in the samples were identified according to the chromatographic and mass spectrometric conditions of each amino acid standard (biovina biotech, China), and the content of the amino acids was calculated.

### Effect of exogenous amino acids on BmNPV replication

L-Arginine and L-Serine were procured from Sigma-Aldrich, USA. These amino acids were individually dissolved in insect Grace medium. BmN cells at a density of 1.25 × 10^5^ cells were seeded in 24-well plates and left to attach. BmN cells were infected with 1 MOI of BmNPV for 1 h at 28°C. The supernatant was then removed and replaced with fresh insect Grace medium containing 10% FBS, supplemented with either serine (5 mM) or arginine (3 mM). The time at which the supernatant was replaced was designated as 0 hpi. Cells and supernatants were collected at 24, 48, and 72 hpi. Targeting the viral capsid gene *vp39*, the intracellular viral mRNA level was quantified using qPCR, while 50% endpoint viral titers in the supernatant were determined using the Reed-Muench method.

### Overexpression and RNA interference (RNAi)

To assess the impact of the amino acid transporter Slc7a6 (BMSK0000671/ XM_004924899.4) on the replication of BmNPV, we modulated its expression by overexpression and RNAi experiments. Initially, primers for double-stranded RNA (dsRNA) production targeted at *Slc7a6* were designed and synthesized (S1 Data) and dsRNA was produced using the T7 RiboMAX Express RNAi System. Subsequently, BmN cells were seeded in 24 well cell culture plates and 5 μg of dsRNA-Slc7a6 was transfected into BmN cells using FuGENE HD Transfection Reagent (Promega, USA) according to the manufacturer’s protocol, while dsRNA-dsRed was used as a control. Cell samples were collected at 24, 48, 72, and 96 h post-transfection to detect the mRNA expression levels of *Slc7a6*. Finally, after transfection of BmN cells with the same concentration of dsRNA-Slc7a6 for 24 h, the cells were infected with 1 MOI of BmNPV. After collecting cell samples at 24, 48, and 72 hpi, both the relative mRNA expression levels of BmNPV, the viral DNA load and the viral titer were assessed.

To overexpress Slc7a6 in BmN cells, we constructed the overexpression vector pIEX-Slc7a6-V5 (Primers in S1 Data). BmN cells were seeded in 24-well plates, and then 500 ng of the overexpression vector pIEX-Slc7a6-V5 or the empty vector pIEX were transfected into the BmN cells using FuGENE HD reagent. The cells were collected at 24, 48, 72, and 96 h post-transfection, and the protein expression level of external Slc7a6-V5 was analyzed by western. Subsequently, BmN cells were infected with 1 MOI of BmNPV 24 h after transfection with the same concentration of pIEX-Slc7a6-V5 (empty vector pIEX was used as control). Cell samples were collected at 24, 48 and 72 hpi and the relative mRNA expression level of BmNPV and viral DNA load were determined using qPCR. In addition, 50% endpoint viral titers in the supernatant were determined.

### Detection of viral proliferation

To detect the expression level of mRNAs, cell samples were collected from different treatments. Total RNA was then extracted using the Fast2000 kit (Fastagen, China) according to the manufacturer’s instructions. Genomic DNA was removed using the PrimeScript RT kit (TaKaRa, Japan) with gDNA Eraser and RNA was reverse transcribed to obtain cDNA. In order to explore the fold change in gene expression, qPCR was performed with 2 × SYBR qPCR premix (Yeasen Biotechnology, China). The primers for qPCR were listed in S1 Data. All experimental data were obtained using the Bio-Rad real-time fluorescence quantitative PCR system and analyzed using the 2^-ΔΔCt^ method. *BmTIF4A* was used as the reference gene and detection of the viral gene *vp39* was used to evaluate BmNPV replication.

For the determination of the viral DNA load, BmN cell samples from different treatments were collected and total DNA from cell samples was extracted using the SteadyPure Universal Genomic DNA Extraction Kit (Accurate Biology, China). The computational analysis of the viral DNA load was carried out according to the method of Chen *et al*. (2018). Briefly, qPCR was performed using primers specific for *gp41* and 5 ng of DNA as template. The absolute amount of the target gene to assess the BmNPV viral DNA load was calculated by intrapolation into a standard curve obtained by dilutions of an external standard [64].

To measure the viral titer, supernatants from BmNPV -infected cells with different treatments were collected at 24, 48, and 72 hpi. Cells were then seeded into 96 -well plates. After the cells adhered to the plates, the collected supernatant samples were serially diluted by 10-fold at each step. Subsequently, 10 μL of each serially the diluted supernatant was added to wells containing 90 μL of BmN cells for incubation. At 72 hpi, green fluorescence in the wells was observed, and TCID_50_ was calculated using the Reed-Muench method [65].

### Western blot

The cell samples were lysed at 4°C for 30 min in a mixture containing the protease inhibitor phenylmethylsulfonyl fluoride (PMSF, 0.5mM). The lysates were then sonicated and the supernatant was collected by centrifugation. The supernatant was mixed with 5 × SDS loading buffer and incubated at room temperature for 30 min to allow denaturation. The samples were separated by SDS-PAGE and transferred to a polyvinylidene fluoride (PVDF) membrane. After blocking for 1 h, the membrane was incubated with V5 mouse monoclonal antibody (Invitrogen, USA), α-Tubulin Rabbit Monoclonal Antibody (Beyotime Biotechnology, China) or ATG8 rabbit polyclonal antibody (AtaGenix Biologicals Ltd, China) at 4°C overnight. Subsequently, the membrane was washed with 1 × TBST buffer (Tris buffered saline with Tween-20) and incubated with horse radish peroxidase (HRP)-labeled Goat anti-Mouse IgG (H + L) or Goat anti-Rabbit IgG (H + L) (Beyotime Biotechnology, China) at room temperature for 1 h. Finally, the antibody-antigen complexes were visualized using enhanced chemiluminescence (Bio-Rad, USA) and Gel XR gel imager (Bio-Rad, USA).

### Construction of knockout cell lines

To further understand the effect of amino acid transporters on BmNPV replication, we constructed a *Slc7a6* knockout (KO) BmN cell line using the CRISPR/Cas9 method [66]. Initially, a guide RNA (gRNA) targeting a specific site on the *Slc7a6* mRNA was designed utilizing the CRISPRdirect online tool (https://crispr.dbcls.jp/). The synthesized gRNA primers were then annealed to form oligo dimers, which were subsequently inserted into the pSL1180-Cas9-U6-sgRNA vector containing eGFP cassette, *Bombyx* U6 promoter and gRNA scaffold [67]. The constructed vector, harboring the gRNA targeting the *Slc7a6* mRNA site, was transfected into BmN cells. After 72 h, green fluorescence was observed, and cells were selected with 350 mg/L Zeocin. Two weeks later, BmN cells were harvested for the extraction of RNA and genomic DNA. PCR was carried out using primers that flank the CRISPR/Cas9 target site using genomic DNA as template. PCR fragments were subsequently checked for mutations in the *Slc7a6* gene. RNA was used for qPCR to determine the mRNA expression levels of *Slc7a6.* Finally, this cell line was designated as Slc7a6^KO^ Cell. The Slc7a6 knockout cells used in Mitochondrial autophagy assay were generated using the same method as the Slc7a6^KO^ cells, but with a knockout plasmid that does not contain the green fluorescent protein eGFP. Therefore, this cell line was designated as Slc7a6^KO-non-eGFP^ cells. (gRNA sequences and primers shown in S1 Data).

### Measurement of arginine concentration in BmN wild-type and Slc7a6^KO^ cells

The arginine content of cell samples from different treatment groups was determined according to the instructions of the arginine content assay kit (Solarbio, China). Briefly, after counting of the number of cells, cell samples were centrifuged at 500 g. To the cell pellets, 1 mL of extraction reagent I were added, and then ultrasonication was performed at 4°C (power 300 W, ultrasonic 3 s, interval 7 s, 3 min totally). Supernatants of cellular extracts were obtained after centrifugation at 12000 g for 10 min at 4°C. To 800 μL of supernatant, 150 μL of the extraction reagent II was added and mixed slowly followed by re-centrifugation at 12000 g for 10 min at 4°C. Final supernatants were added to the analytical solution for the determination of arginine by absorbance at 525 nm (colorimetric assay).

### Regulation of autophagy by treatment of BmN cells with inhibitors/inducers

To investigate whether BmNPV infection of host cells regulates arginine content in host cells through autophagy, we used the autophagy inhibitor Trichostatin A (TSA) (MedChem Express, USA) and the autophagy inducer C646 (MedChem Express, USA), which have been studied to regulate the onset of autophagy in *B. mori* [24,68]. First, cytotoxicity was assessed using the CCK-8 assay kit (Beyotime Biotechnology, China). Specifically, Slc7a6^KO^ cells were grown in 96-well plates, followed by treatment of the cells with different concentrations of C646 or TSA for 24 hours. Afterwards, cell culture medium containing 10% CCK-8 reagent was added to the BmN cells and incubated at 28°C for 2 h to determine cell viability. The optimal concentration was considered the highest concentration at which no toxic effects were observed in the cells. The corresponding cells were first pretreated with the drugs for 12 h, and then the cells were incubated with 1 MOI of BV for 1 h. Cells were collected at 24 hpi, 48 hpi and 72 hpi and the expression of ATG8-PE was detected by Western blotting.

To investigate the effect of autophagy changes on the changes of intracellular arginine content and the proliferation level of BmNPV, cells were treated with the optimal concentration of TSA or C646 as described above, and after infection with BmNPV, cells were collected to detect the changes of arginine in the cells and the changes of BmNPV proliferation.

### Mitochondrial autophagy assay

Previous studies have shown that BmNPV infection leads to mitochondrial damage [24]. The occurrence of mitochondrial autophagy during BmNPV infection was monitored using the Mito-QC reporting system [25]. In brief, we constructed a recombinant overexpression plasmid, pIEX-mCherry-eGFP-Fis1, which includes the tandem mCherry-eGFP tag fused to the mitochondrial targeting sequence of the silkworm mitochondrial fission 1 protein, Fis1 (BMSK0002319). After transfection of 1 μg of the pIEX-mCherry-eGFP-Fis1 plasmid into cells, cells were infected with BmNPV at 1 MOI. At 72 hpi, green and red fluorescence signals were observed, photographed and compared using a laser confocal microscope (Leica SP8, Germany). Fiji software (version 2.3) was used for green fluorescence statistics. To understand the role of mitochondrial autophagy during BmNPV infection, we used the mitochondrial autophagy inhibitor Cyclosporin A (CSA) (MedChem Express, USA) at the determined optimal concentration (S4C Fig) to perform experiments using the same treatments as TSA and C646.

### Statistical analysis

Three independent replications of all experiments were performed (virus titer assay experiments were performed at least twice) to ensure the reliability of the results. The results of the experiments are presented as mean ± standard deviation (Mean ± SD). For analyzing the differences between the different groups, we used GraphPad Prism software (version 9.0) to perform Two-Way ANOVA tests to determine the significance of the variances. If the *P*-value was less than 0.05, the results were considered statistically significant. For other types of experiments, we used an unpaired *t*-test to assess statistical significance, again using a *P*-value of less than 0.05 as the criterion.

## Supporting information

S1 Fig Changes in the levels of nonessential amino acids in hemolymph following BmNPV infection. (TIF)

S2 Fig Serine promotes BmNPV replication. (A, B) After pretreatment of BmN cells with 5 mM serine for 12 h and incubation of the cells with 1 MOI of BmNPV, cell and supernatant samples were collected at 24, 48, and 72 hpi. The mRNA level of the viral gene *vp39* was detected by qPCR (A) and the viral titer by TCID_50_ determination (B).
(TIF)

S3 Fig Characterization of Slc7a6 knockout cell lines. (A) Green fluorescence of BmN cells after transfection with pSL1180-Cas9-U6-sgRNA vector containing eGFP cassette. Scale bar: 100 μm. (B) Detection of mRNA levels of Slc7a6 in Slc7a6^KO^ cells and WT cells. (C) Cell viability of Slc7a6^KO^ cells, as assessed by the CCK8 assay. (D) Levels of arginine in Slc7a6^KO^ cells infected with BmNPV at 24, 48, and 72 h.
(TIF)

S4 Fig Cytotoxicity assay for drug-treated cells. (A) Slc7a6^KO^ cells were treated with different concentrations of TSA (1 nM, 2 nM, 4 nM, and 8 nM) as well as DMSO (Solvents for TSA) for 24 h. Subsequently, cytotoxicity was detected using the CCK8 kit. The cytotoxicity tests indicate that BmN cells can be treated with TSA at 4 nM without toxicity. (B) Slc7a6^KO^ cells were treated with different concentrations of C646 (100 nM, 200 nM, 400 nM, and 600 nM) as well as DMSO (Solvents for C646) for 24 h. Subsequently, cytotoxicity was detected using CCK8 kit. The cytotoxicity tests indicate that BmN cells can be treated with C646 at 100 nM without toxicity. (C) Slc7a6^KO^ cells were treated with different concentrations of CSA (10 nM, 20 nM, 30 nM, 40 nM, and 50 nM) as well as DMSO (Solvents for CSA) for 24 h. Subsequently, cytotoxicity was detected using CCK8 kit. The cytotoxicity tests indicate that BmN cells can be treated with CSA at 30 nM without toxicity.
(TIF)

S5 Fig BmNPV infection induces autophagy in Slc7a6^KO^ cells. (A) 1 μg of the pIEX-mCherry-eGFP-Fis1 plasmid was transfected into BmN cells. At 24 hpi, the cells were incubated with BmNPV at MOI of 1 for 1 h. After the incubation period, the cells were washed and cultured in fresh medium. At 72 hpi, green and red fluorescence changes in the cells were observed using a laser confocal microscope (Mito-QC reporting system). Scale bar: 100 μm and 5 μm. (B) Calculation of green fluorescence intensity of mitochondria in cells using Fiji software.
(TIF)

S1 Data Primers used in the study. (DOCX)
