# Supplementary figures and images for: Baculovirus enhances arginine uptake and induces mitochondrial autophagy to promote viral proliferation
Source: PLoS Pathog. 2025 Jul 8;21(7):e1013331. doi: 10.1371/journal.ppat.1013331 (PMC12273963; doi:10.1371/journal.ppat.1013331)

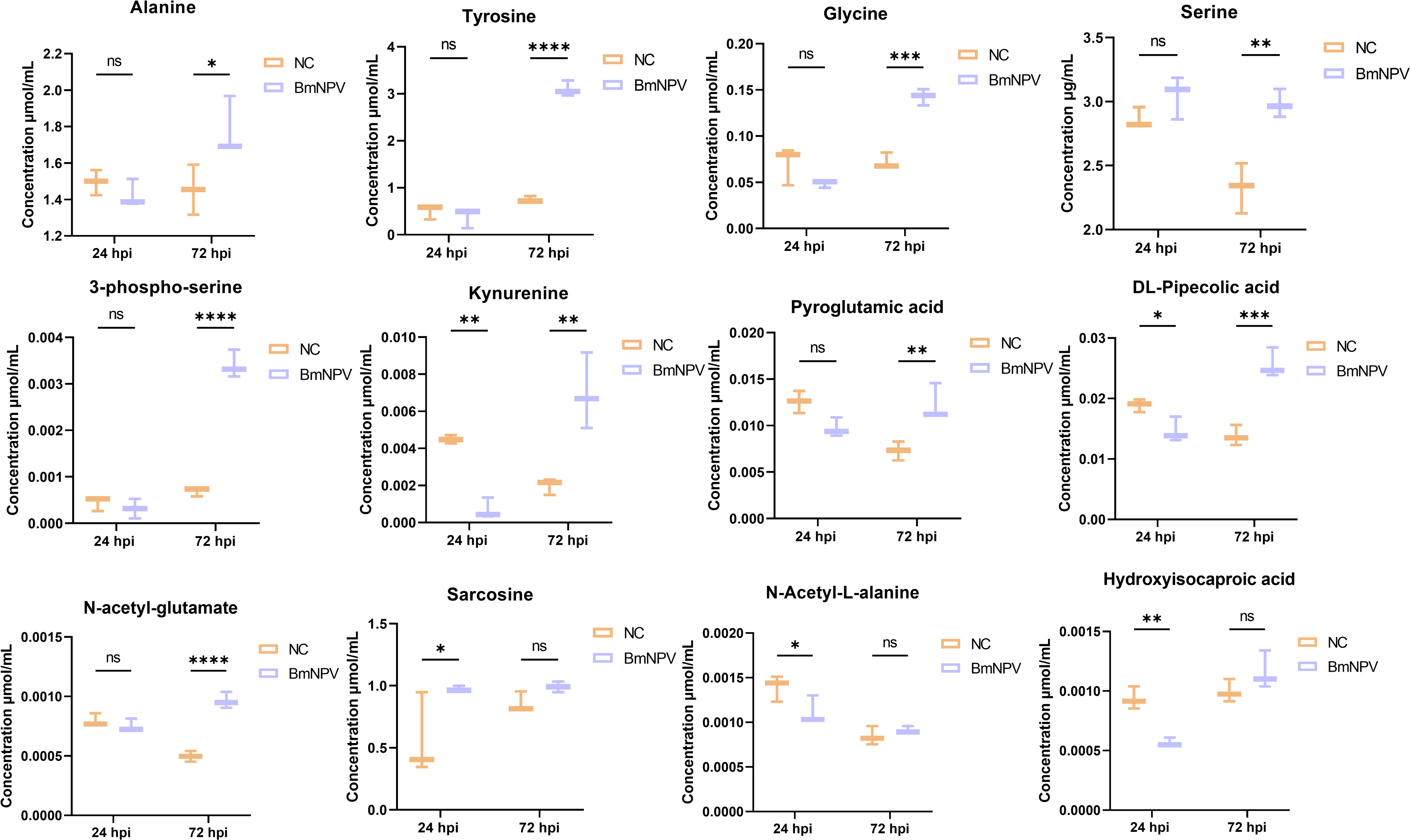

Supplement: S1 Fig — (TIF) [file ppat.1013331.s001.tif]

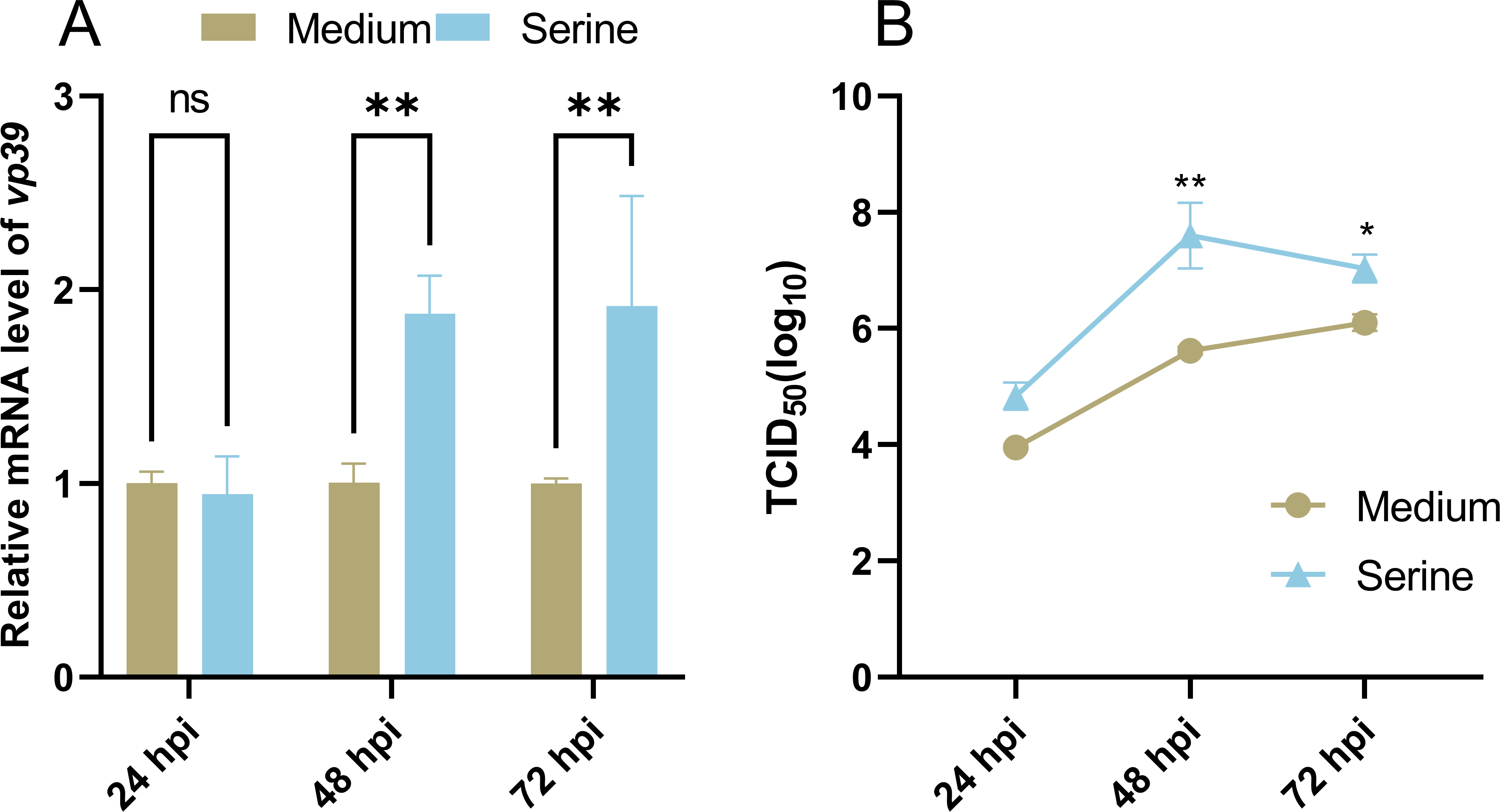

Supplement: S2 Fig — (A, B) After pretreatment of BmN cells with 5 mM serine for 12 h and incubation of the cells with 1 MOI of BmNPV, cell and supernatant samples were collected at 24, 48, and 72 hpi. The mRNA level of the viral gene vp39 was detected by qPCR (A) and the viral titer by TCID50 determination (B). (TIF) [file ppat.1013331.s002.tif]

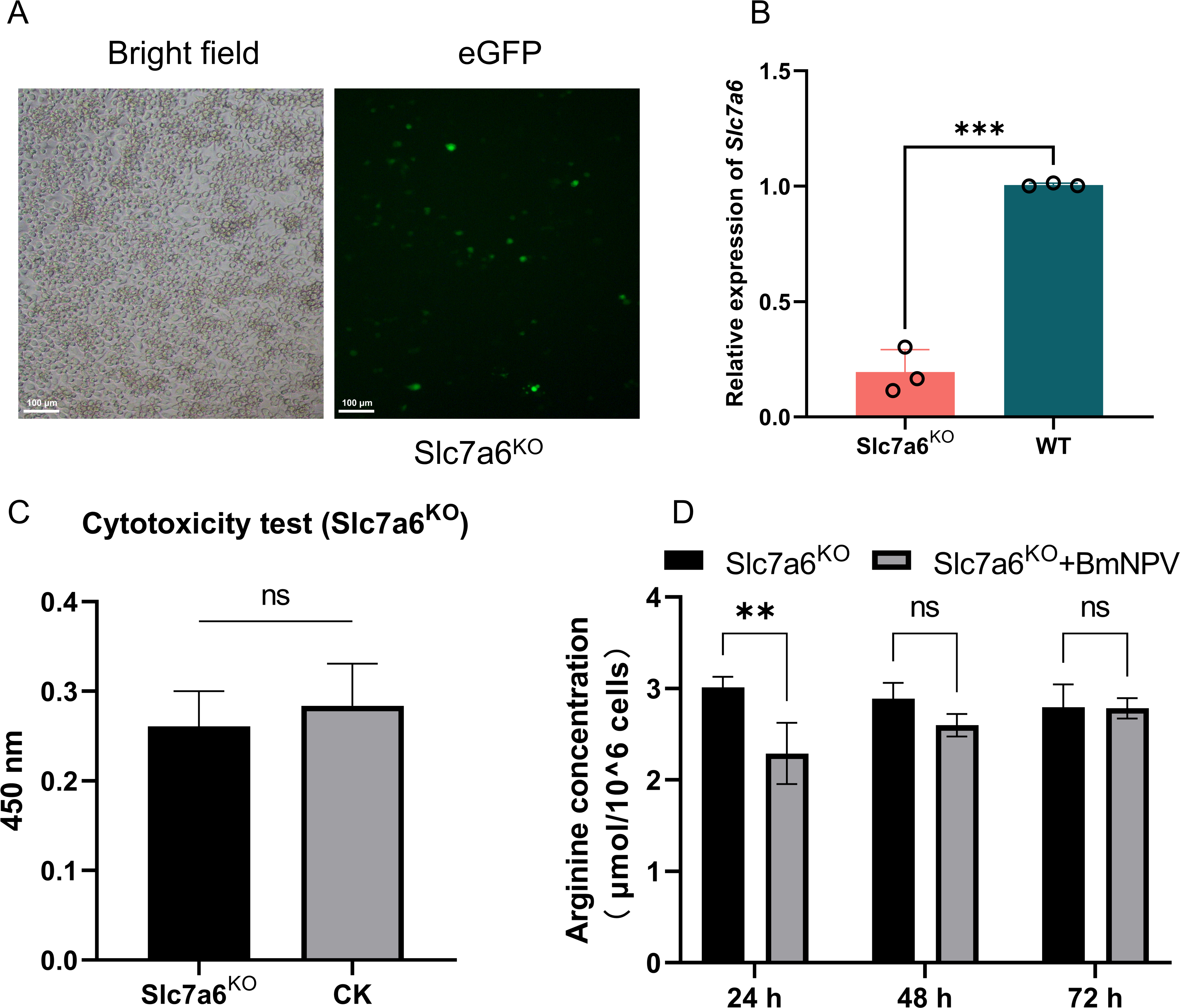

Supplement: S3 Fig — (A) Green fluorescence of BmN cells after transfection with pSL1180-Cas9-U6-sgRNA vector containing eGFP cassette. Scale bar: 100 μm. (B) Detection of mRNA levels of Slc7a6 in Slc7a6KO cells and WT cells. (C) Cell viability of Slc7a6KO cells, as assessed by the CCK8 assay. (D) Levels of arginine in Slc7a6KO cells infected with BmNPV at 24, 48, and 72 h. (TIF) [file ppat.1013331.s003.tif]

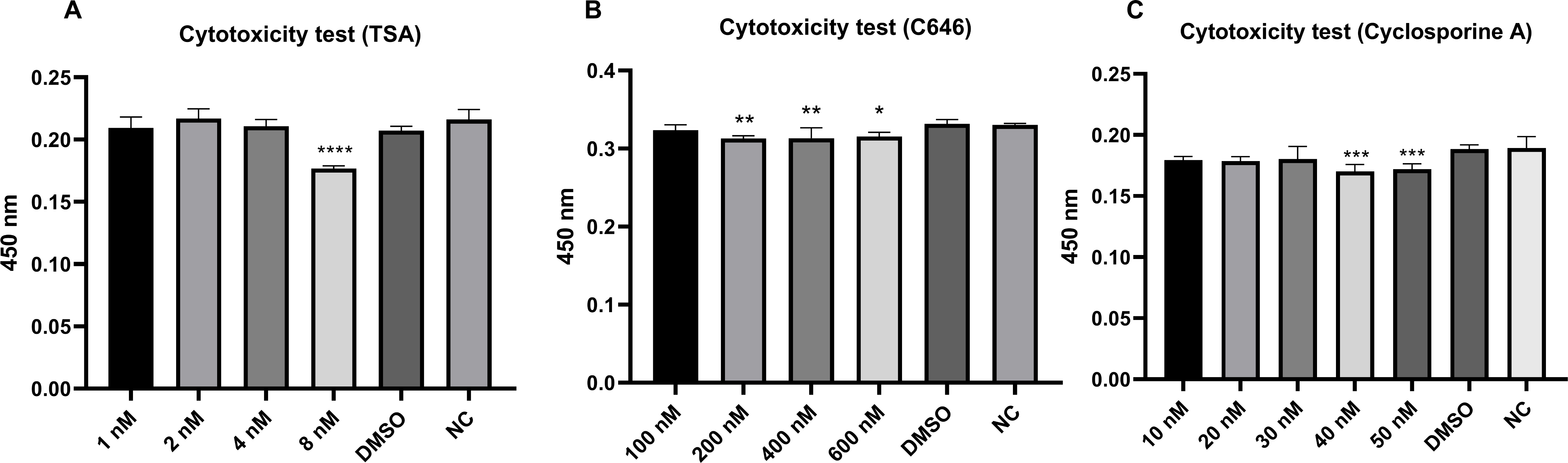

Supplement: S4 Fig — (A) Slc7a6KO cells were treated with different concentrations of TSA (1 nM, 2 nM, 4 nM, and 8 nM) as well as DMSO (Solvents for TSA) for 24 h. Subsequently, cytotoxicity was detected using the CCK8 kit. The cytotoxicity tests indicate that BmN cells can be treated with TSA at 4 nM without toxicity. (B) Slc7a6KO cells were treated with different concentrations of C646 (100 nM, 200 nM, 400 nM, and 600 nM) as well as DMSO (Solvents for C646) for 24 h. Subsequently, cytotoxicity was detected using CCK8 kit. The cytotoxicity tests indicate that BmN cells can be treated with C646 at 100 nM without toxicity. (C) Slc7a6KO cells were treated with different concentrations of CSA (10 nM, 20 nM, 30 nM, 40 nM, and 50 nM) as well as DMSO (Solvents for CSA) for 24 h. Subsequently, cytotoxicity was detected using CCK8 kit. The cytotoxicity tests indicate that BmN cells can be treated with CSA at 30 nM without toxicity. (TIF) [file ppat.1013331.s004.tif]

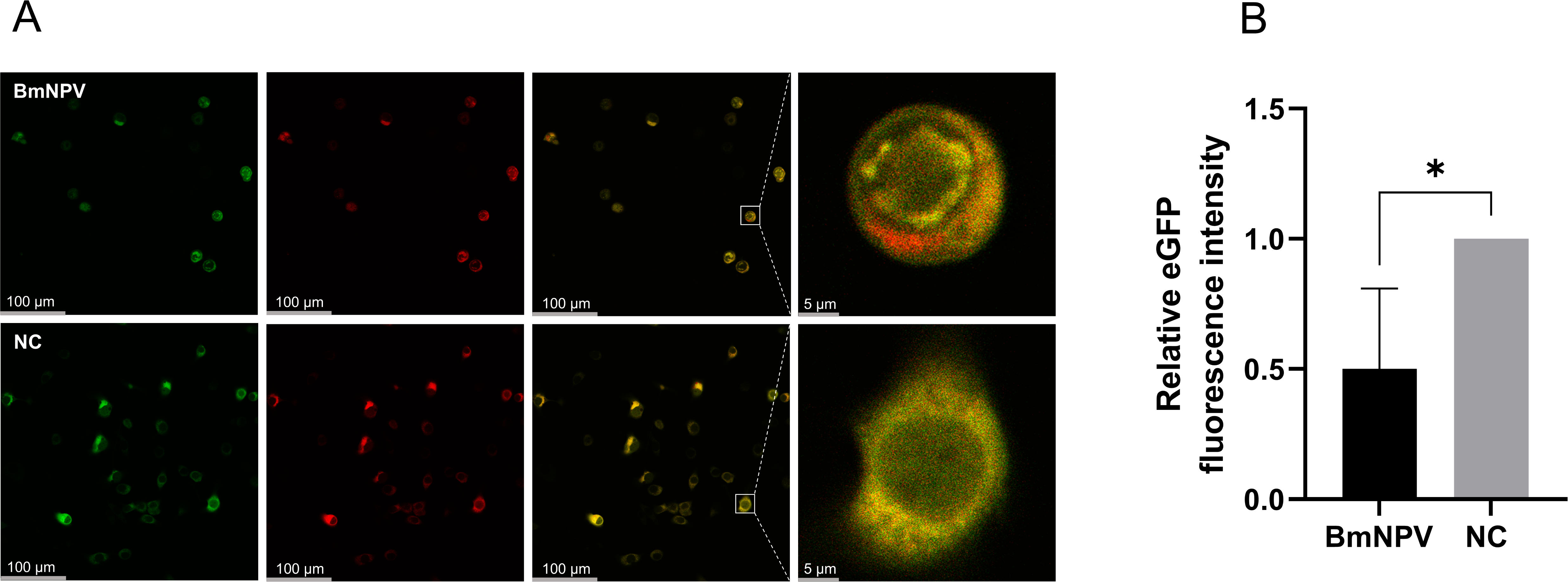

Supplement: S5 Fig — (A) 1 μg of the pIEX-mCherry-eGFP-Fis1 plasmid was transfected into BmN cells. At 24 hpi, the cells were incubated with BmNPV at MOI of 1 for 1 h. After the incubation period, the cells were washed and cultured in fresh medium. At 72 hpi, green and red fluorescence changes in the cells were observed using a laser confocal microscope (Mito-QC reporting system). Scale bar: 100 μm and 5 μm. (B) Calculation of green fluorescence intensity of mitochondria in cells using Fiji software. (TIF) [file ppat.1013331.s005.tif]
