# Supplementary material for: Baculovirus enhances arginine uptake and induces mitochondrial autophagy to promote viral proliferation
Source: PLoS Pathog. 2025 Jul 8;21(7):e1013331. doi: 10.1371/journal.ppat.1013331 (PMC12273963; doi:10.1371/journal.ppat.1013331)
Supplement: S1 Data — (DOCX) [file ppat.1013331.s006.docx]

Table S1 Primers used in the study.

| Purpose | Gene name | Sequence of Oligonucleotide (5’-3’) |
| --- | --- | --- |
| RNAi  (dsRNA) | Slc7a6 | F: taatacgactcactatagggCTTCGGAAGTGGGATTTTCA |
|  |  | R: taatacgactcactatagggTTTCAAAATGCTCTGTGCCA |
|  | DsRed | F: taatacgactcactatagggGAAGCTGAAGGTGACCAAGG |
|  |  | R: taatacgactcactatagggTGGTGTAGTCCTCGTTGTGG |
| qPCR | Slc7a6 | F: GAGAGGTGGCACAGAGCATT |
|  |  | R: AATGCCACAGACCACTCAGC |
|  | TIF4A | F: GAATGGACCCTGGGACACTT |
|  |  | R: CTGACTGGGCTTGAGCGATA |
|  | vp39 | F: CTAATGCCCGTGGGTATGG |
|  |  | R: TTGATGAGGTGGCTGTTGC |
|  | gp41 | F: ATGTTGATGTGCGGAAAGC |
|  |  | R: GTGGCGGAATCGGTGA |
| over-  expression | Slc7a6 | F: AACAACCAAGTGACCATGGGCATGGTCGAAGAAAGAATAAAAATGCG |
|  |  | R1: GAGGAGAGGGTTAGGGATAGGCTTACCTTCTTCCTTGTCTTCGA |
|  |  | R2: ATTGGGATCCGCGAGCTCCTACGTAGAATCGAGACCGAGGAGAGGGTTA |
|  | Fis1 | F: GGGGGTGGAGGCTCTATGGAAGACGTATTA |
|  |  | R: ATTGGGATCCGCGAGCTCCTATTTCTTTGA |
|  | mCherry | F: AACAACCAAGTGACCATGGGCATGGTG |
|  |  | R: CACCATGCTAGCCTTGTACAGCTC |
|  | eGFP | F: GAGCTGTACAAGGCTAGCATGGTGAGCAAG |
|  |  | R: GTCTTCCATAGAGCCTCCACCCCCCTTGTACAGC |
| gRNA | Slc7a6 | CTCGCTGTACTGGGACCAGT |
| Primers for knockout cell assays | Slc7a6 | F: CCCAACACGGCATAATAATATCCTAA |
|  |  | R: CTTTAAACATATATGAAGCAAGGGTGTAAC |

The wavy line represents the sequence of the T7 promoter.
